# Proton pump inhibitors (PPIs) impact on tumour cell survival, metastatic potential and chemotherapy resistance, and affect expression of resistance-relevant miRNAs in esophageal cancer

**DOI:** 10.1186/s13046-014-0073-x

**Published:** 2014-09-01

**Authors:** Kirsten Lindner, Christiane Borchardt, Maren Schöpp, Anja Bürgers, Christian Stock, Damian J Hussey, Jörg Haier, Richard Hummel

**Affiliations:** 1 Department of General and Visceral Surgery, Muenster University Hospital, Waldeyerstr. 1, Muenster, 48149, Germany; 2 Institute of Physiology, University of Muenster, Muenster, Germany; 3 Department of Surgery, Flinders Medical Centre, Flinders University, Adelaide, Australia; 4 Comprehensive Cancer Centre, University of Muenster, Muenster, Germany

**Keywords:** Proton pump inhibitor, PPI, Esophageal cancer, Metastasis, Chemotherapy, Resistance, microRNA

## Abstract

**Background:**

Neoadjuvant treatment plays a crucial role in the therapy of advanced esophageal cancer. However, response to radiochemotherapy varies widely. Proton pump inhibitors (PPIs) have been demonstrated to impact on chemotherapy in a variety of other cancers. We analyzed the impact of PPI treatment on esophageal cancer cell lines, and investigated mechanisms that mediate the effect of PPI treatment in this tumour.

**Methods:**

We investigated the effect of esomeprazole treatment on cancer cell survival, adhesion, migration and chemotherapy in human adeno-(OE19) and squamous-cell-carcinoma (KYSE410) cell lines. Furthermore, we investigated the effect of PPI treatment on intra-/extracellular pH and on expression of resistance-relevant miRNAs.

**Results:**

Esomeprazole significantly inhibited tumour cell survival (in a dose-dependent manner), adhesion and migration in both tumour subtypes. Furthermore, esomeprazole augmented the cytotoxic effect of cisplatin and 5-FU in both tumour subtypes. Surprisingly, PPI treatment led to a significant increase of intracellular pH and a decrease of the extracellular pH. Finally, we found esomeprazole affected expression of resistance-relevant miRNAs. Specifically, miR-141 and miR-200b were upregulated, whereas miR-376a was downregulated after PPI treatment in both tumour types.

**Conclusion:**

Our study demonstrates for the first time that PPIs impact on tumour cell survival, metastatic potential and sensitivity towards chemotherapy in esophageal cancer cell lines. Furthermore, we observed that in this tumour entity, PPIs do not lead to intracellular acidification, but affect the expression of resistance-relevant miRNAs.

## Background

Esophageal cancer is one of the most fatal malignancies in the world, with a dramatic increase in incidence in the western world, especially of the adenocarcinoma subtype [1]. Despite improvements in the management of esophageal cancer patients, the general outcome remains very poor for both histological subtypes, with an overall 5-year survival of approximately 10% and a 5-year post-esophagectomy survival rate of approximately 15-40% [2],[3]. The main reason lies in the lack of early clinical symptoms, which usually results in advanced tumour stages at the date of diagnosis. In an attempt to improve outcome of patients after surgery, and to potentially increase the number of patients who qualify for surgery by reducing the size of the primary tumour, neoadjuvant therapy is used. Several recent meta-analyses have demonstrated the potential of neoadjuvant therapy in advancing overall survival for both histological subtypes, particularly for therapy responders. Additionally, tumour reduction and nodal ¿down-staging¿ were described as independent prognostic factors for better outcome after neoadjuvant therapy [3]¿[9]. Furthermore, in un-resectable disease, chemotherapy and irradiation showed good results, with complete tumour regression in up to 50% of patients and partial response in approximately 25% of patients. Therefore, cisplatin- and 5-fluorouracil (5-FU)-based chemotherapy in combination with irradiation has become part of standard treatment in neoadjuvant, definitive and palliative settings in most parts of the world [10]¿[12].

However, the resistance of tumours to anticancer drugs such as cisplatin or 5-FU is a major obstacle in the non-surgical anticancer treatment of esophageal cancer. One potential mechanism that confers chemotherapy resistance is disruption of the pH gradient. Hypoxic conditions in tumour cells are often observed during the development of solid tumours, leading to intracellular and extracellular acidosis [13]. This change of intra- and extracellular pH may impair the uptake of weakly basic chemotherapeutic drugs and reduce their effects on tumours [13]¿[15]. Recent studies demonstrated that proton pumps such as vacuolar adenosine triphosphatases (V-ATPases) are involved in tumour invasion and multi-drug-resistance in breast cancer [16],[17], oral squamous cell carcinoma [18],[19], hepatocellular carcinoma [20], pancreatic cancer [21] and prostate cancer [22]. Further, there is accumulating evidence in the literature that chemotherapy resistance of various tumours can be reduced via so called proton pump inhibitors (PPIs) that disrupt the pH gradient by inhibition of proton pumps [23]¿[25]. PPI pretreatment has been shown to sensitize various cell lines derived from primary tumours, including colon and ovarian adenocarcinomas, to cisplatin, 5-FU and vinblastine [26]. Most interestingly, there is some evidence suggesting that high concentrations of PPIs alone can induce apoptosis in gastric and hepatoblastoma cancer cell lines but not in non-tumourous primary cells [27],[28].

However, to the best of our knowledge, there is no data available on PPIs as potential antitumour agents or modulators of drug resistance in esophageal cancer. In this context, we were interested if proton pump inhibitors such as esomeprazole might potentially serve as a new first-line drug or as an additive to currently available chemotherapeutics in the treatment of esophageal cancer. Specifically, we aimed to investigate 1) if PPI treatment impacts on tumour cell survival, metastatic potential and drug resistance of esophageal squamous cell carcinoma and adenocarcinoma cell lines, and if yes: 2) which cellular mechanisms mediate the effect of PPIs on tumour cells.

## Methods

### Cell lines and cell cultures

The human esophageal squamous cell carcinoma (SCC) cell line KYSE410 and the human esophageal adenocarcinoma (EAC) cell line OE19 were selected for our study. Cells were cultured using RPMI 1640 medium (GIBCO® Invitrogen, #11875), supplemented with 10% fetal bovine serum (GIBCO® Invitrogen, #26140), 1% Penicillin-Streptomycin (GIBCO® Invitrogen, #15140; 10.000 units of penicillin and 10.000 ?g of streptomycin per ml) and 2% Normocin¿ (InvivoGen, San Diego USA, Catalog # ant-nr-1; 50 mg/ml) in a humidified atmosphere containing 5% CO_2_ at 37°C. For functional assays and chemotherapy experiments, phenol red free medium (RPMI 1640: GIBCO® Invitrogen, #11835) containing the same supplements were used. Cells were cultured using standard techniques and reagents [10],[29]. All experiments were carried out in at least 3 technical replicates and 3 independent experiments unless otherwise stated.

### Proton pump inhibitor treatment with esomeprazole for functional analyses

For viability assays, cells were plated onto 96-well plates and allowed to attach for 24 hours (SCC) or 48 hours (EAC). Then, phenol red free medium containing esomeprazole (Nexium®, AstraZeneca, Germany) at various concentrations was freshly prepared and added to the corresponding cells. After 72 hours, cell viability assays were performed as described below.

For adhesion and migration assays, cells were incubated in T75 flasks for 72 hours with esomeprazole at the approximate median lethal doses (LD50, as estimated from cell viability experiments). Adhesion and migration assays were then performed as described below.

For chemotherapy experiments, cells were treated for 72 hours with either esomeprazole alone at different concentrations (50 ?M: ¿sub-lethal¿, 86-100% cell survival; 250 ?M: ¿lethal¿, 20-30% cell survival; 350 ?M: ¿highly lethal¿, <10% cell survival), or with cisplatin or 5-fluorouracil at the LD50 concentrations, or with esomeprazole and chemotherapeutics together.

For experiments on the effect of PPI treatment on intra- and extracellular pH/proton concentrations or on miRNA expression, cells were incubated for 24/48/72 respectively 72 hours with esomeprazole at the approximate LD50 dosis (as estimated from cell viability experiments). Experiments were then performed as described below.

### Cell viability assay

Cell viability was assessed using MTT (Thiazolyl Blue Tetrazolium Bromide, Sigma-Aldrich, St. Louis, USA: no. M2128). 100 ?l MTT solution (1 mg/ml MTT in cell culture medium) was added per well. After three hours, the supernatant was removed and the MTT formazan crystals were solubilized for 30 minutes in 100 ?l dimethyl sulfoxide (Sigma-Aldrich) per well. Finally, the absorbance at 570 nm was measured on the spectrophotometer Dynatech MR5000 (Dynatech, Ashford, UK) using the software MikroWin 2000 (Mikrotek Laborsysteme, Overath, Germany).

### Adhesion assay

Cells in T75 flasks were incubated for 72 hours with esomeprazole at the approximate LD50 dose, and subsequently underwent a 75 minute starving period using serum free medium. Cells were trypsinized and incubated for 90 minutes for reconstitution, then cells were transferred to 96-well plates coated with collagen type I and fibronectin. These cells were plated under the stimulation of TGF-?2, and cellular adhesion was assessed after 15/30/60/90 minutes under the photospectrometer using crystal violet staining. One experiment was performed with 4 technical replicates, and confirmed with another independent experiment.

### Migration assay

Cells in T75 flasks were incubated for 72 hours with esomeprazole at the approximate LD50 dose, and subsequently underwent a 3 hour starving period using serum free medium. They were plated onto the upper chamber of a 24-well Boyden chamber coated with collagen type I and/or fibronectin (Corning B.V. Life Sciences, Amsterdam, The Netherlands; Cat. No. 3428) with an 8-?m pore polycarbonate membrane in medium without serum, and medium containing 10% fetal bovine serum was filled in the lower chamber as chemoattractant. After 12 hours, cells that did not migrate through the pores were removed using cotton swabs. Membranes were stained using crystal violet, and migrating cells were counted in 9 gridded high-power fields per membrane under an inverted microscope. One experiment was performed with 3 technical replicates, and confirmed with another independent experiment.

### Chemotherapeutic treatment

Cells were seeded onto 6-well plates (9.5×10^4^ viable cells/well for KYSE410 and 2×10^5^ viable cells/well for OE19) and allowed to attach. After reaching 10-20% confluence, fresh medium containing EITHER no PPI and chemotherapeutics OR esomeprazole or chemotherapeutics alone OR esomeprazole and chemotherapeutics together was prepared and added to the corresponding cells. Regarding the different esomeprazole doses used in these experiments please see above. The concentrations of chemotherapeutics used represented the approximate LD50 doses after 72 hour exposure (OE19: 25 ?M cisplatin, 20 ?M 5-FU; KYSE410: 7.5 ?M cisplatin, 20 ?M 5-FU; determined in previous experiments, data not shown). After 72 hour exposure, cell viability assays were performed as described above in order to assess the impact of isolated or combined treatment with esomeprazole and chemotherapeutics on cell survival. In addition cells were lysed using TRIzol® reagent (Invitrogen Life Technologies, NY, USA) according to the instructions of the manufacturer, and stored at ?80°C for later RNA processing as described previously [10].

### Measurement of intra- and extracellular pH

Intracellular pH (pHi) was measured using video imaging techniques and the fluorescent pH indicator (2-carboxyethyl)-5- carboxyfluorescein (BCECF)-AM (Molecular Probes, Eugene, OR, USA) according to the manufacturer¿s protocol. Briefly, the pH value of solution A was adjusted to 7.43, 7.05 and 6.50 and the pH value of solution B was adjusted to 7.4. Nigericin was diluted with ddH2O at 5 mM (3.375 mg Nigericin:1 ml ddH2O). 1 ?l Nigericin solution was added into 1 ml solution A with the final concentration of 5 mM. BCECF-AM pH-sensitive fluorescent probe was diluted into 5 mM with DMSO and stored at ?20°C away from light. Cells were cultured for 24, 48 or 72 hours on glass-bottom-dishes (35 mm diameter, Greiner Bio-One) with and without esomeprazole (LD50), at a density of 1×10^5^ cells per dish for KYSE410 and 3,8x10^5^ cells per dish for OE19, in cell culture medium as mentioned above. Then, the medium was replaced with 2 ml solution B and the cells were incubated in a humidified atmosphere containing 5% CO_2_ at 37°C. 2.5 ?g/ml BCECF was added directly to the dishes and cells were incubated for 5 minutes. Thereafter, the glass bottom dish was continuously superfused with 37°C HEPES-buffered Ringer solution. pHi was measured using BCECF fluorescence. BCECF was excited with light of 440 nm and 490 nm wavelengths. The emitted fluorescence intensities were measured at 37°C in intervals of 25 seconds and monitored at the 500 nm wavelength using a Photometrics camera (CoolSnap*fx*, Visitron Systems, Puchheim, Germany). A high-speed polychromator system (Visichrome, Visitron Systems) was used to generate the different wavelengths. Polychromator and data acquisition were controlled by the software MetaFluor (Visitron Systems). Finally the measurements of each experiment were calibrated by successively replacing the HEPES-buffered Ringer solution with modified Ringer solutions of pH 7.4, 7.0 and 6.5, each containing 10 ?mol/l Nigericin (Sigma-Aldrich), to determine the pHi. Per glass bottom dish, the pHi of at least 20 single cells within the field of view was measured. Three independent experiments were performed with KYSE410 and OE19, respectively.

For extracellular pH measurement cells were grown in 6 well plates (Sarstedt) at an initial density of 1.9?×?10^5^ (KYSE410) or 3.8?×?10^5^ (OE19) viable cells per flask for 72 hours during esomeprazole pre-treatment (LD50). Extracellular pH (pHe) of the culture medium was then measured after 72 hours of PPI treatment by pH211 Calibration Check Microprocessor pH Meter (Seven Multi Mettler Toledo, Germany).

### Analysis of changes in expression of resistance-relevant miRNAs after PPI treatment

For assessment of a potential impact of PPI treatment on miRNA expression, 18 miRNA were selected from our own previous work (manuscript accepted). Briefly, we conducted experiments with cisplatin- and 5-FU resistant EAC and SCC cell lines and investigated the miRNA expression pattern of these resistant cell lines. From the miRNAs that showed differential expression between resistant and sensitive cell lines, we then selected 18 miRNA candidates that, according to current literature, have an important impact on chemotherapy resistance in cancer cells. For the purpose of this study, we refer to these miRNAs as ¿resistance-relevant¿. Namely, we selected miR-16, miR-21, miR-23a, miR-24, miR-26a, miR-106, miR-141, miR-155, miR-196a, miR-200a, miR-200b, miR-200c, miR-221, miR-222, miR-296-5p, miR-376a, miR-429 and let-7i for this study.

The miScript PCR system (Qiagen, Germany) was then used to analyze miRNA expression of the resistance relevant miRNA candidates after PPI treatment (LD50). miScript assays were performed according to the manufacturer¿s instructions. Briefly, for each sample, 500 ng of DNase pre-treated RNA was used for reverse transcription into cDNA. Following the manufacturer¿s protocol, we utilized 4 ?l miScript 5X RT Buffer, 1 ?l Reverse Transcriptase and 5 ?l nuclease-free water. Incubation of reagents was performed in a thermocycler (protocol: 60 minutes at 37°C, 5 minutes at 95°C, then a hold at 4°C). For real-time PCR, 2 ?l of cDNA was mixed with 10 ?l QuantiTect SYBR, 2 ?l 10X miScript Universal Primer, 2 ?l gene specific 10X miScript Primer Assay, and 4 ?l nuclease-free water. All samples were assayed in triplicate reactions using a BioRad CFX 384 Real-Time System (Hercules/California USA). Quantitative analysis was performed using Bio-Rad CFX Manager 2.1. MiRNA expression data were normalized to the expression levels of SNORD25, SNORD44 and SNORD68, which displayed comparable expression across the different groups (data not shown).

### Statistical analysis

All data are means?±?standard deviation unless otherwise stated. The relative cell survival after PPI treatment (viability assay) and after treatment with anticancer drugs was calculated by normalizing the mean corrected absorbance of the treated cells to the corresponding untreated controls (given in%). For assessment of the effect of PPI treatment on sensitivity to chemotherapy, the relative survival of the negative controls was then be set to ¿0¿, and the effect of pre-treatment was presented as relative survival of treated cells compared to negative controls (given in%). Data were assessed for statistical significance using parametric (Student¿s *t*-test for equal and unequal variances) tests as appropriate. P <0.05 was considered to be statistically significant. All analyses were performed using SPSS 20.0 (SPSS, Chicago, IL).

## Results

### Esomeprazole inhibits survival of esophageal cancer cell lines

**Figure 1:**
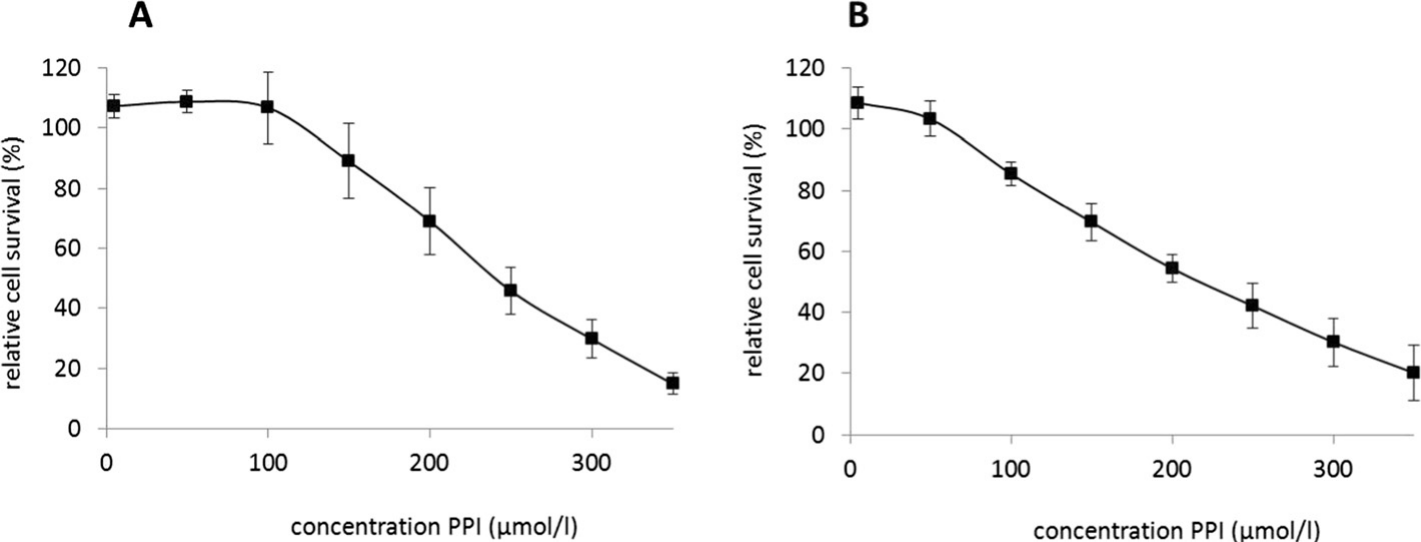
**Dose¿response curve of PPI treatment in esophageal cancer cell lines.** The figure presents an overview of the impact of PPI treatment with esomeprazole on tumour cell survival in SCC **(A)** and EAC **(B)** cells. PPI: proton pump inhibitor esomeprazole.

At first, we aimed to assess if esomeprazole impacts on survival of esophageal cancer cell lines. Figure 1 presents an overview of the dose¿response curves of PPI treatment with esomeprazole at various doses in SCC (A) and EAC (B) cell lines. In both tumour subtypes, increasing esomeprazole doses were dose-dependently associated with decreasinging cell survival with increasing esomeprazole doses, thus providing evidence for a negative impact of PPI treatment on tumour cell survival. In this context, sublethal doses were approximately 50 ?M in both cell lines, and LD50 of SCC and EAC cells were 250 ?M and 200 ?M, respectively.

### Esomeprazole suppresses the metastatic potential of esophageal cancer cell lines

**Figure 2:**
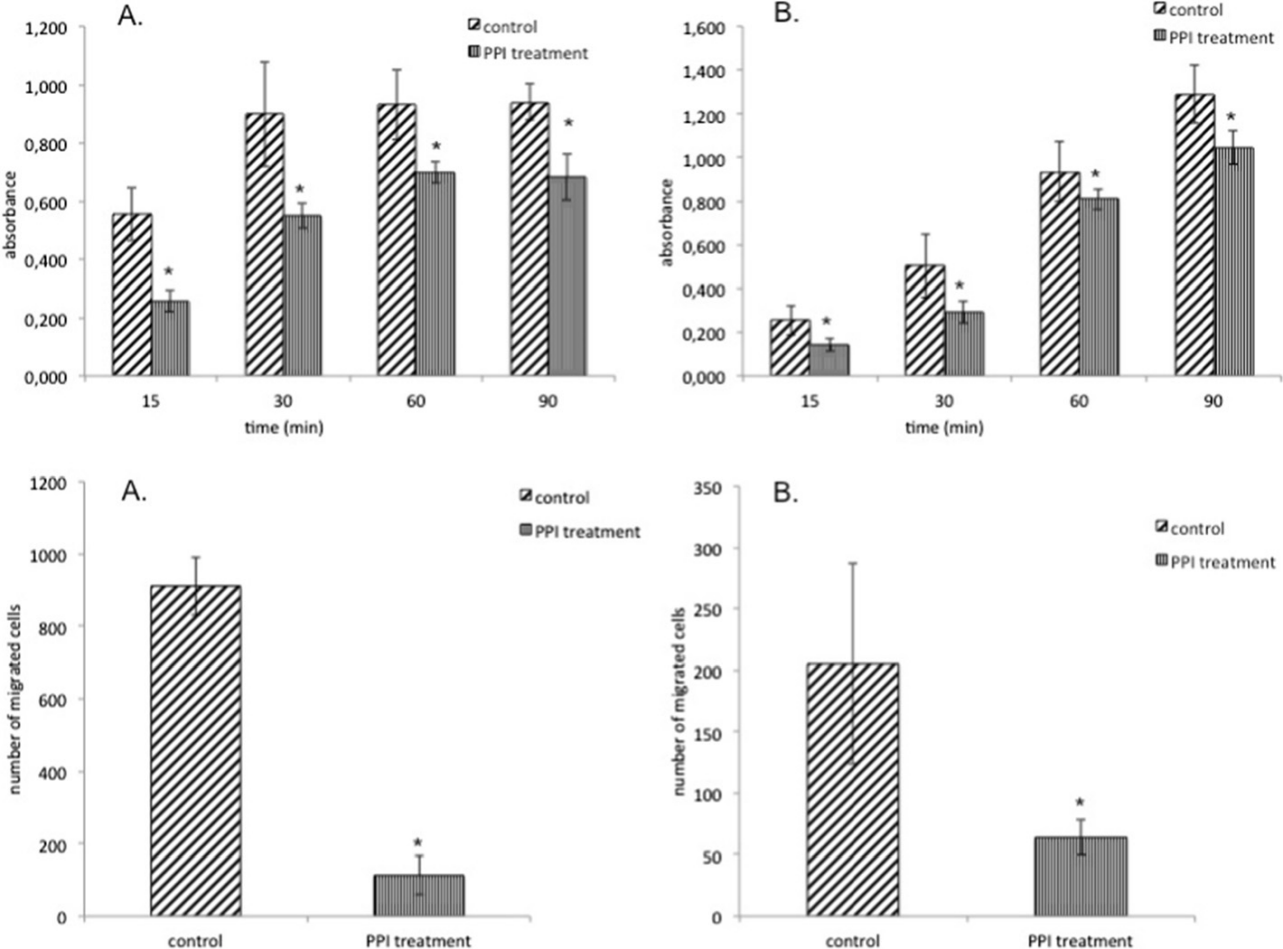
**Effect of PPI treatment on metastatic potential of esophageal cancer cell lines.** The figure presents an overview about the effect of PPI treatment on cell adhesion (1) and migration (2) in SCC **(A)** and EAC **(B)** cell lines. Negative controls (i.e. adhesion and migration assays with uncoated wells) were performed though for visual clarity they are not included in the figures. PPI treatment: treatment with proton pump inhibitor esomeprazole. Control: untreated control cells. *: statistically significant different compared to control (p???0.025).

Adhesion and migration are key determinants of the ability of tumour cells to metastasize into distant organs, as metastasis includes invasion of circulating tumour cells into distant organs where the tumour cells have to adhere and migrate through the endothelium of the vessels. We therefore investigated the impact of esomeprazole treatment on adhesion and migration in esophageal cancer cell lines. Figure 2 presents an overview of the results of adhesion and migration assays performed on SCC (A) and EAC (B) cell lines after PPI treatment with esomeprazole. After 15, 30, 60 and 90 minutes of PPI treatment, the ability of tumour cells to adhere to coated wells under the stimulation of TGF-?2 was significantly reduced in both tumour entities compared to untreated controls (p???0.025). Furthermore, the ability of tumour cells (SCC and EAC) to migrate through 8-?m pores in a coated Boyden Chamber was significantly reduced after PPI treatment compared to controls (p?<?0.0001).

### Esomeprazole augments the cytotoxic effect of cisplatin and 5-FU in esophageal cancer cell lines

**Figure 3:**
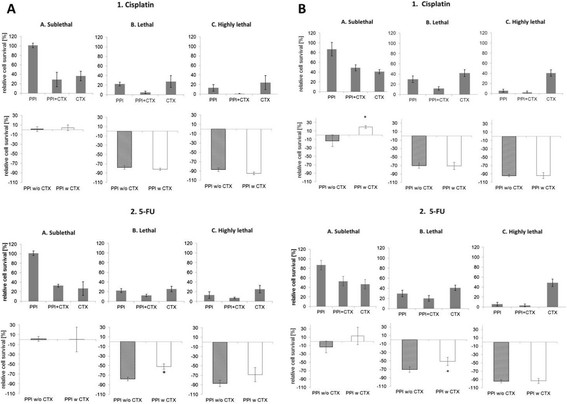
**Effect of PPI treatment on otherwise untreated cells and on CTX treated cells.** Presents an overview of the impact of esomeprazole treatment on otherwise untreated cells or on cells that were treated simultaneously with chemotherapeutics **(3A: SCC; 3B: EAC)**. Tumour cells were treated with either esomeprazole alone at different concentrations (50 ?M: ¿sub-lethal¿, 86-100% cell survival; 250 ?M: ¿lethal¿, 20-30% cell survival; 350 ?M: ¿highly lethal¿, <10% cell survival), or with cisplatin or 5-FU at the respective LD50 concentrations, or with esomeprazole and chemotherapeutics together. The upper graphs present an overview of the relative cell survival of the respective groups (PPI treated cells versus chemotherapy (CTX) treated cells versus PPI?+?CTX treated cells). The lower graphs present an overview about the additional cytotoxic effect of PPI treatment on otherwise untreated cells (PPI w/o CTX) or on CTX treated cells (PPI w CTX). PPI: proton pump inhibitor esomeprazole. CTX: chemotherapy. *: statistically significant different compared to control.

Given the suppressive effect of esomeprazole on the survival and metastatic potential of esophageal cancer cells, we were interested if esomeprazole might affect the sensitivity of esophageal cancer cells towards commonly used chemotherapeutic drugs such as cisplatin and 5-FU. We therefore treated tumour cells with either esomeprazole alone at different concentrations, or with cisplatin or 5-FU at the respective LD50 concentrations, or with esomeprazole and chemotherapeutics together. Figure 3 presents an overview of the impact of esomeprazole treatment on otherwise untreated cells or on cells that were treated simultaneously with chemotherapeutics. Esomeprazole in ¿sub-lethal dose¿ did not impact on survival of untreated or simultaneously chemotherapy treated SCC or EAC cancer cells. Applied in ¿lethal¿ or ¿highly lethal doses¿, however, esomeprazole reduced the survival of otherwise untreated cells of both tumour entities (p?<?0.05) as expected. Most interestingly, at these doses, esomeprazole presented a significant additional cytotoxic effect on cells treated with cisplatin or 5-FU in both SCC and EAC cells (p?<?0.05). However, this additional effect of esomeprazole on the cytotoxicity of chemotherapeutics was higher in cisplatin treated cells (resulting in an overall cytotoxicity of 88-99% after combined treatment) than in 5 FU-treated cells (resulting in an overall cytotoxicity of only about 80-97% after combined treatment; p?<?0.05).

### Esomeprazole does not lead to intracellular acidification and extracellular alkalisation in esophageal cancer cell lines

**Figure 4:**
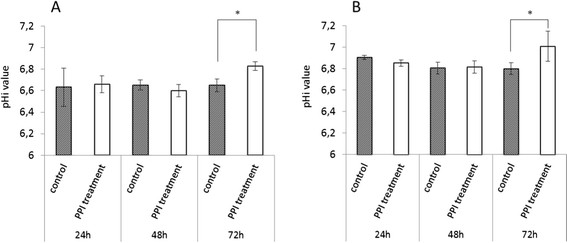
**Effect of PPI treatment on intracellular pH.** The figure presents the results of intracellular pH measurement after 24/48/72 hours of esomeprazole treatment (LD50) in SCC **(A)** and EAC **(B)** cells. PPI treatment: treatment with esomeprazole. *: statistically significant different compared to control (p???0.017).

**Figure 5:**
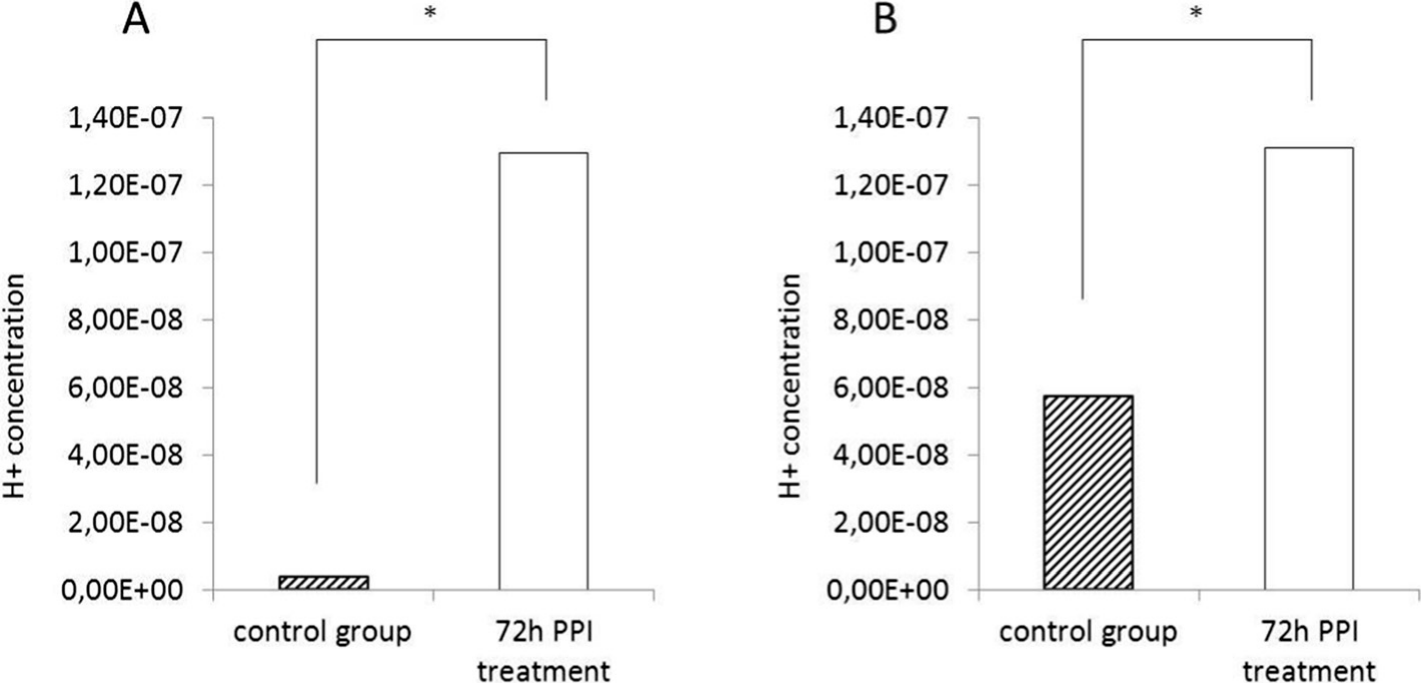
**Effect of PPI treatment on extracellular proton concentration.** The figure presents the concentration of protons in the extracellular space (culture medium) after 72 hour PPI treatment (LD50) in SCC **(A)** and EAC **(B)** cells. *: statistically significant different compared to control (p???0.001).

The literature suggests that PPIs mediate their effects on tumour cells via disruption of the intra-extracellular pH-gradient and accumulation of protons in the cytosol of cancer cells. We hypothesized that the observed suppressive effect of esomeprazole on cell survival, metastatic potential and sensitivity towards cisplatin and 5-FU in both esophageal cancer subtypes might be caused by intracellular acidification/extracellular alkalisation. Therefore, we investigated the intracellular pH in both tumour subtypes, and the proton concentration in the extracellular space (culture medium). We could not detect any differences in the intracellular pH between cells that were exposed to esomeprazole (LD50) for 24/48 hours and untreated controls. However, surprisingly, the intracellular pH was significantly higher in cells (SCC and EAC) treated with esomeprazole for 72 hours compared to untreated controls (p???0.017). In addition, the concentration of protons was significantly higher in the extracellular space of esomeprazole treated cells (72 hours, LD50) compared to untreated controls (p???0.001) (see Figures 4 and 5).

### Esomeprazole affects expression of resistance-relevant miRNAs

**Figure 6:**
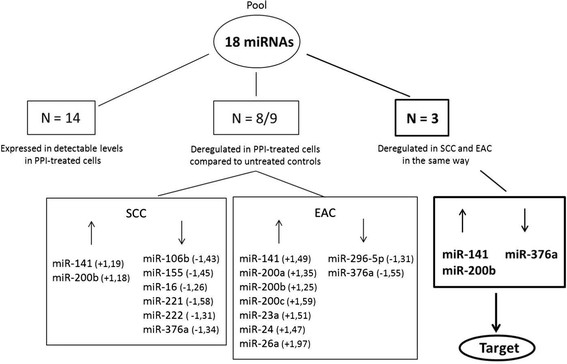
**Effect of PPI treatment on expression of resistance-relevant miRNAs.** The figure presents an overview about the significant deregulation of selected miRNAs in SCC and EAC cells in after treatment with esomeprazole (LD50) for 72 hours, compared to controls. ?: significant upregulation. ?: significant downregulation.

miRNAs are important epigenetic regulators of tumour cell survival, metastatic potential and sensitivity towards chemotherapeutic drugs. We hypothesized that esomeprazole might mediate its effects on these factors via regulation of miRNAs. Therefore, we selected 18 miRNAs that we previously found to be resistance-relevant, and assessed their expression pattern in esomeprazole treated cells and untreated controls. From these 18 miRNAs, 14 candidates were expressed at detectable levels in the tumour cells. After PPI treatment, we observed significant deregulation of 8 of these miRNAs in SCC cells and 9 of these miRNAs in EAC cells. Most interestingly, 3 of these resistance-relevant miRNA candidates showed a similar pattern of deregulation in both tumour types: miR-141 and miR-200b were significanty upregulated whereas miR-376a was significantly downregulated (see Table 1 and Figure 6).

**Table 1:** Effect of PPI treatment on expression of resistance-relevant miRNAs

**miRNAs**	**SCC**	**EAC**
miR-16	?1,26?±?0,12	/
miR-23a	/	+1,51?±?0,20
miR-24	/	+1,47?±?0,17
miR-26a	/	+1,97?±?0,3
miR-106	?1,43?±?0,14	/
miR-141	+1,19?±?0,07	+1,49?±?0,16
miR-155	?1,45?±?0,09	/
miR-200a	/	+1,35?±?0,05
miR-200b	+1,18?±?0,08	+1,25?±?0,11
miR-200c	/	+1,59?±?0,09
miR-221	?1,58?±?0,17	/
miR-222	?1,31?±?0,26	/
miR-296-5p	/	?1,31?±?0,29
miR-376a	?1,34?±?0,16	?1,55?±?0,08

The table presents an overview of the significant deregulation/fold-change in expression of selected miRNAs in SCC and EAC cells after treatment with esomeprazole (LD50) compared to controls. +: significant upregulation. -: significant downregulation.

## Discussion

The overall prognosis of esophageal cancer patients remains very poor. However, conservative treatment options, especially neoadjuvant radiochemotherapy, have been widely adopted because they provide a benefit for overall survival in patients with locally advanced disease and good response to neoadjuvant treatment [3]¿[6],[8],[9]. However, only a subset of patients presents a major response to radiochemotherapy, and only these patients finally profit from this therapeutic option [4],[5],[7],[30]. This problem highlights the urgent need for new treatment options, either as first-line treatment, or as additive treatment to conservative therapies that improves the effect of radiochemotherapy. In this context, proton pump inhibitors (PPIs) might provide a new tool for treatment of esophageal cancer. Based on the highly promising results in other tumour entities [19],[23]¿[25], we hypothesized that PPIs might impact on tumour cell survival, metastatic potential and chemotherapy resistance in esophageal cancer.

Our data provide the first evidence that the proton pump inhibitor esomeprazole has cytotoxic effects on esophageal cancer cell lines, by suppressing cell survival of SCC and EAC cell lines, in a dose-dependent manner. Furthermore, we found that esomeprazole inhibits adhesion and migration, two key aspects of tumour metastasis, in SCC and EAC cell lines. This supports the conclusion that PPIs reduce the metastatic potential of esophageal cancer cells. We also demonstrated that esomeprazole has an additive effect on the cytotoxicity of the commonly used chemotherapeutics, cisplatin and 5-FU, in both histological subtypes. Taken together, our results demonstrate for the first time that PPIs such as esomeprazole have an effect on tumour cell survival, metastatic potential and sensitivity towards different chemotherapeutics in esophageal cancer cell lines, as has previously been reported in other tumour entities. This highlights their potential use as first-line treatment option or additive therapy in combination with chemotherapy in esophageal cancer patients.

On the search for cellular mechanisms that mediate the effect of esomeprazole on esophageal cancer cells, we first focussed on the potential of PPIs to disrupt the intra-extracellular pH gradient. This was described as the main mechanism of action of PPIs in other malignancies such as prostate cancer [23], breast cancer [24], colon cancer [26] and ovarian cancer [26]. However, most surprisingly, we detected that esomeprazole treatment led to an intracellular increase of pH in both SCC and EAC cells after 72 hour of treatment. Furthermore, the concentration of extracellular protons was higher after 72 hour PPI treatment compared to untreated controls. This observation does not support the hypothesis that in esophageal cancer cells, PPIs mediate their effects mainly via inhibition of membrane based proton pumps and subsequent acidification of the intracellular space and alkalisation of extracellular space. In contrast, our experiments suggested that PPI treated cells were still able to eliminate protons from the intracellular space and to (at least in part) excrete them into the extracellular compartment. Therefore, we hypothesized that esomeprazole might mediate its impact on esophageal cancer cells via epigenetic regulation. We found that esomeprazole treatment leads to deregulation of a number of chemotherapy resistance-relevant miRNAs. Specifically, PPI treatment led to upregulation of miR-141 and miR-200b and downregulaton of miR-376a in SCC and EAC cells.

Our results on the effects of the PPI ,esomeprazole, on tumour cell survival, metastasis and chemotherapy resistance in esophageal cancer are supported by current literature on other tumour entities. For example, it was described that proton pump inhibitors can induce apoptosis or inhibit tumour cell growth in gastric or hepatoblastoma cancer cell lines but not in non-tumourous primary cells at high concentrations [27],[28]. Oral administration of a small molecule inhibitor of V-ATPase, NiK-12192, was reported to cause a significant inhibition of formation of spontaneous metastases of a human lung tumours in nude mice [31]. Furthermore, several studies reported that V-ATPases are involved in tumour invasion and multi-drug-resistance in many types of cancer [16]¿[22]. In addition, a number of authors demonstrated an effect of PPIs or other V-ATPase inhibitors on cancer treatment. For example, PPIs were shown to increase the sensitivity of colon adenocarcinoma derived cells towards chemotherapeutic drugs [32], or specific inhibitors of V-ATPase were demonstrated to impair the preferential accumulation of daunomycin in lysosomes and to reverse the resistance towards anthracyclines in drug-resistant renal epithelial cells [33]. In a screening study of small molecules that disturbed the anti-apoptotic function of Bcl-2 or Bcl-xL, Sasazawa and coworkers found that V-ATPase inhibitors such as bafilomycin A1 were able to induce apoptosis in drug resistant cells following treatment with taxol [34]. Further evidence for the role of V-ATPases in chemoresistance was reported from targeted molecular studies: small interfering RNA against the ATP6L subunit of proton pump V-ATPase was shown to attenuate chemoresistance of breast cancer cells [16] and hepatocellular carcinoma xenografts [20].

Regarding the effect of PPI treatment on intra- and extracellular pH, our data are somewhat contradictory to most reports in the current literature. Tumours were reported to present an intracellular pH ranging from 7.12 to 7.56 (pHi of normal cells: 6.99-7.20), and an extracellular pH of 6.2-6.9 (pHe of normal extracel- lular space: 7.3-7.4), which is controlled by key pH regulators that maintain a neutral/alkaline intracellular pH by eliminating lactate or protons. Extracellular acidity in tumours tends to be associated with a poorer prognosis based on its effect on aggressiveness, metastasis and resistance towards chemotherapy and radiotherapy treatment [35]. Proton pumps such as V-H ATPases play a key role in the control of the intra-extracellular pH-gradient. These pumps are ATP-dependent membrane-based transporters that control pHi and pHe by actively transport protons from the cytoplasmic compartment to the extracellular space or into other intracellular vesicles [36]. Consequently, inhibition of proton pumps via PPIs should impair the ability of the cell to eliminate protons from the intracellular space, subsequently leading to an accumulation of protons in the cytosol of the cells with a decrease of pHi, and an increase of pHe. And in fact, Chen et al. observed a decrease of pHi and an increase of pHe in a human gastric cell line after PPI treatment [32]. Moreover, Luciani and colleagues demonstrated that PPI pretreatment of melanoma, colon adenocarcinoma, breast cancer and ovarian carcinoma cell lines was associated with an increase of both, pHe and the pH of lysosomal organelles [26]. Furthermore, there is some evidence in the current literature that changes in pHi and pHe impact on prognosis [37], invasiveness and metastasis formation [38], activation of extracellular metalloproteases that influence tumour cell motility, proliferation and metastasis [23], and resistance towards irradiation and chemotherapy drugs [35]. For example, acidic pHe in tumours can lead to an extracellular accumulation of weakly basic chemotherapeutics such as anthracyclines, anthraquinones and vinca alkaloid which subsequently fail to reach their intracellular targets. Thus, an increased extracellular acidity in tumours can promote multi drug resistance [23]¿[25].

In contrast to these reports, we found that pHi increased and pHe decreased after PPI treatment in esophageal cancer cell lines. We acknowledge that this different effect of PPI treatment on pHi and pHe might be influenced by specific biological characteristics which separate esophageal cancer from other tumour entities. However, our data provide the first reported evidence that in esophageal cancer cell lines, PPI treatment does not lead to an intracellular accumulation of protons and an inability to eliminate protons in the extracellular compartment. Our data suggest that the observed effect of PPI treatment in our study might at least in part not be mediated by the inhibition of proton pumps.

Regarding a potential effect of PPI treatment on expression of miRNAs, our study shows for the very first time that esomeprazole treatment impacts on expression of resistance-relevant miRNAs. There are no prior reported studies that investigate the potential of PPIs to alter miRNA expression in either SCC or EAC. Most interestingly, we found three miRNAs (namely miR-141, miR-200b and miR-376a) to be deregulated in a similar fashion in both tumour subtypes, implying that these miRNAs might in general be affected by PPI treatment. Furthermore, all three miRNAs have been previously described to impact on tumour cell survival and chemotherapy resistance in various cancer types. Imanaka et al. reported that miR-141 was highly expressed in cisplatin-resistant SCC cell lines [39], and van Jaarsveld and colleagues found an association between miR-141 levels and response to cisplatin therapy in ovarian cancer patients [40]. In addition, elevated miR-200b levels were described to influence cell proliferation, invasion and migration in gastric cancer [41], and the development of multi drug resistance in Ehrlich asites cell lines [42]. In prostate cancer cells, miR-376 was shown to be involved in regulation of proliferation, apoptosis, migration and cell invasion [43]. Furthermore, this miRNA was also found to be involved in multi drug resistance [44].

There are a few limitations of the current study that have to be considered for proper interpretation of our results. Firstly, the current study represents an in-vitro study with only one esophageal adenocarcinoma and one squamous cell carcinoma cell line. This means that our data cannot be immediately transferred into clinical settings, as results might be limited to the selected cell lines and reproducibility might be limited. However, this is the first study that investigates the effect of PPI treatment on esophageal cancer, and we selected well known and commonly used esophageal cancer cell lines. Therefore, in our opinion this data provides a valid basis for further investigations in additional in-vitro or in-vivo experiments. Secondly, we used esomeprazole doses of up to 250 ?M in our experiments. In this context, maximal tissue concentrations after esomeprazole administration in humans have to be considered in order to achieve clinically relevant data on the effect of esomeprazole on tumour characteristics. Based on product information from Astra Zeneca, 40 mg i.v. esomeprazole (which is the standard dose of esomprazole per day in the therapy of peptic ulcer and gastritis) would achieve a steady state tissue concentration of 6 ?M for an 80 kg human. However, in specific situations such as hypersecretory conditions, recommended adult oral starting dose of esopmeprazole is 60 mg once daily with subsequent adjustment of individual doses, and doses up to 120 mg three times daily have been administered. The doses used in our experiments are higher than the currently clinically used doses. However, PPIs are considered to be generally safe in application. Despite some reported adverse side effects such as osteoporosis and bone fracture, hypomagnesaemia, the development of gastric polyps, enteric infections, interstitial nephritis and pneumonia, and the absolute risk of complications attributed to PPIs is low [45]. Moreover, the doses used in our experiments are similar to those of other research groups [14]. Thirdly, we did not include an analysis of the expression pattern of proton pumps in the cell membrane or in membranes of intracellular vesicles, or of the exact percentage and strength of inhibition of the proton pumps via esomeprazole. We only analysed the intra- and extracellular pH and concluded from these data that both cell lines were still able to excrete protons into the extracellular space. However, as several other authors observed that PPI treatment lead to intracellular acidification, in our opinion the absence of this accumulation of protons in the intracellular space in our experiments justifies the conclusion that this is not the main effect of action of esomeprazole in esophageal cancer cell lines. Finally, we selected a number of resistance-relevant miRNAs from our previous experiments on drug resistant cell lines, and did not include screening of general miRNA expression pattern after PPI treatment, or target validation. This approach would allow a more sophisticated interpretation of the effect of PPI treatment on miRNA expression. However, our experiments aimed to simply investigate if miRNA deregulation caused by PPI treatment might be a potential mechanism for the impact of PPI treatment on cancer cells. We showed that esomeprazole altered expression of a number of miRNAs that are well known to impact on tumour cell survival and drug resistance in the current literature.

## Conclusion

The current study provides for the very first time evidence that PPIs impact on tumour cell survival, metastatic potential and sensitivity towards chemotherapeutic drugs in esophageal cancer cell lines, as has previously been demonstrated in other malignancies. Unexpectedly, we observed that in esophageal cancer cell lines PPI treatment does not lead to intracellular acidification and extracellular alkalisation, factors previously described, in other tumour entities, as a potential mechanism for decreased aggressiveness and drug resistance of tumours after PPI treatment. Most interestingly, we found, that the expression of resistance-relevant miRNAs in esophageal cancer cells (SCC and EAC) is affected by PPI treatment. miRNAs are key players in the epigenetic control of global gene expression, and the effect of PPIs on miRNA expression which we observed may be a previously unrecognised mechanism of PPI action on tumours. Our study provides an important step towards developing a new therapeutic approach for esophageal cancer, especially as PPIs are already widely used in the clinic and do not exhibit major side effects. However, further in-vitro and in-vivo experiments are needed to determine if PPIs can be used as either first-line treatment or additive therapy in esophageal cancer patients.

## Competing interests

The authors declare that they have no competing interests.

## Authors¿ contributions

RH, DJH, JH and KL conceived and designed the experiments and designed the manuscript. AB performed the functional analyses; AB and MS performed the chemotherapeutic treatment; CB and CS performed the pH measurement; CB performed the real time-PCR. RH, JH and KL analyzed data. KL, DJH and RH wrote the manuscript with support of the other authors. All authors read and approved the final manuscript.
